# A microfluidic device to study neuronal and motor responses to acute chemical stimuli in zebrafish

**DOI:** 10.1038/srep12196

**Published:** 2015-07-21

**Authors:** Raphaël Candelier, Meena Sriti Murmu, Sebastián Alejo Romano, Adrien Jouary, Georges Debrégeas, Germán Sumbre

**Affiliations:** 1 Sorbonne Universités, UPMC Univ. Paris 06, UMR 8237, Laboratoire Jean Perrin, F-75005 Paris, France; 2 CNRS UMR 8237, Laboratoire Jean Perrin, F-75005 Paris, France; 3 Ecole Normale Supérieure, Institut de Biologie de l’ENS, IBENS, Paris, France; 4 INSERM, U1024, 75005 Paris, France; 5 CNRS, UMR 8197, 75005 Paris, France

## Abstract

Zebrafish larva is a unique model for whole-brain functional imaging and to study sensory-motor integration in the vertebrate brain. To take full advantage of this system, one needs to design sensory environments that can mimic the complex spatiotemporal stimulus patterns experienced by the animal in natural conditions. We report on a novel open-ended microfluidic device that delivers pulses of chemical stimuli to agarose-restrained larvae with near-millisecond switching rate and unprecedented spatial and concentration accuracy and reproducibility. In combination with two-photon calcium imaging and recordings of tail movements, we found that stimuli of opposite hedonic values induced different circuit activity patterns. Moreover, by precisely controlling the duration of the stimulus (50–500 *ms*), we found that the probability of generating a gustatory-induced behavior is encoded by the number of neurons activated. This device may open new ways to dissect the neural-circuit principles underlying chemosensory perception.

One of the main goals in neuroscience is to understand how the nervous system detects, processes and converts sensory stimuli into appropriate motor patterns. To do so, it is necessary to finely control the spatiotemporal patterns of sensory stimuli while monitoring the dynamics of large neural networks in awake animal models. Among vertebrate models, zebrafish offers a unique combination of assets for such studies. The transparency and relatively small dimensions of the brain at the larval stage, and the availability of transgenic lines expressing genetically encoded calcium indicators offer the possibility to perform functional imaging at single-cell resolution of virtually the whole brain in intact behaving animals^1^,^2^,^3^,^4^.

The ability to deliver complex stimuli yet precisely controlled and with spatio-temporal structures analogous to those encountered in natural conditions is currently met only for the visual and auditory systems. In the zebrafish larva only the visual system was extensively explored where complex sensory-motor behaviors, consistent with that observed in freely swimming organisms, can be elicited by projecting natural scenes in front of an agarose-embedded larva^1^,^5^. In aquatic species chemosensation is used to find food sources^6^, avoid danger^7^, and to swallow or reject ingested particles^8^,^9^. In turbulent environments, akin to rivers, chemicals are transported in the form of fine-scale plumes such that the sequence of stimulation experienced by the fish consists of brief sporadic on/off stimuli. The time sequence of these events have been suggested to carry important information regarding the presence and location of the chemical source^10^.

Delivering precise sequences of sharp pulses of water-soluble chemical stimuli to a tethered larva is a non-trivial problem. Methods that rely on passive transport from a point source inevitably produce a slow and uncontroled increase of the chemical concentration in the vicinity of the fish receptors. More active methods expose the face of the fish to the outlet of a small tube connected to a liquid valve, allowing to sequentially deliver buffer and stimulus solutions. In this configuration, the characteristic switching time is controlled by the tube length and the flow rate. The latter being constrained by the fish dimension and the maximum flow velocity ethologically acceptable, standard macroscopic delivery systems yield switching times in the 1–10 *s* range (see references and discussion in Supplementary information).

Here, we designed and fabricated a microfluidic device capable of delivering multiple chemical stimuli with unprecedented spatial and temporal precision (in the 1–10 *ms* range) to a larva partially restrained in agarose. This stimulation microfluidic device was combined with two-photon functional imaging and high-speed video recording to simultaneously monitor the brain activity and motor behavior. We first performed a comprehensive characterization of the microfluidic device delivery dynamics and demonstrated its capability of inducing taste-specific neuronal responses in the primary gustatory center of teleost fish, the vagal lobe, without generating mechanosensory stimulation. Second, we observed that two gustatory stimuli with different hedonic values (aversive and appetitive) induced distinct activity patterns in the primary gustatory center (vagal lobe) and in higher processing areas (gustatory telencephalon, Dm). Finally, the ability to precisely control the stimulus duration allowed us to study the neuronal principles underlying gustatory-induced motor behaviors. We showed that the stimulus duration (50–500 ms) was positively correlated with the the number of activated neurons in the vagal lobe, the probability of inducing a tail flip and the number of induced tail flips.

## Results

### The Microfluidic device

**Figure 1:**
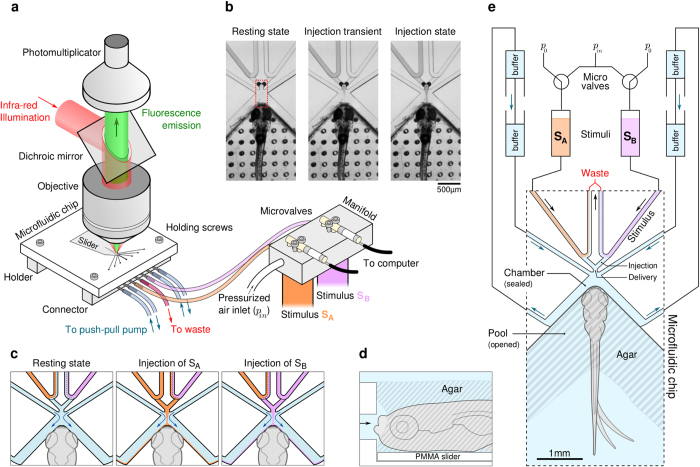
Microfluidic device for precise delivery of chemical stimuli. (**a**) Setup scheme: an epifluorescence two-photon microscope images neural activity while pulse-like stimuli are delivered to a zebrafish larva. An IR-sensitive camera images tail behavior from below. The microfluidic chip is connected to a computer-controlled manifold for the delivery of two different stimuli, *S*_*A*_ and *S*_*B*_. (**b**) Infrared images of the device around the larva’s face during injection of stimulus *S*_*A*_ (infrared dye, left channel). *S*_*B*_ (right channel) is buffer. The red rectangle indicates the region used in the kinematic view of Fig. 2a,b (c) Sketch of the fluid flow in the device during the three stationary states: at rest, during injection of *S*_*A*_ and during injection of *S*_*B*_. (**d**) Side-view scheme of the larva’s head resting on the movable slider. The animal’s face is gently cleared before positioning. (**e**) Scheme of the microfluidic device: the larva’s head embedded in agarose lying on the mobile slider is positioned in the pool in front of the delivery channel. Two electro-microvalves control pressures in stimuli reservoirs *S*_*A*_ and *S*_*B*_ and trigger injection. *p*_0_ is the atmospheric pressure and *p*_*in*_ > *p*_0_.

The microfluidic devices (Fig. 1 and Supp. Fig. 1) were fabricated by micro-milling onto transparent acrylic (PMMA) slabs. The fluidic channels, 100 *μm* in width and 25 to 200 *μm* in height, were sealed by 250 *μm*-thick transparent PMMA sheets using chemically-assisted thermal bonding. The circuit outlets are directed towards an open pool designed such that the larva, partially embedded in agarose, has its mouth precisely positioned in front of the delivery channel (Fig. 1d,e). The microfluidic device consists of two mostly independent circuits. The first one, driven by a push-pull syringe pump, imposes a continuous buffer flow circulating around the larva’s face (blue arrows in Fig. 1e). The second circuit controls the delivery of two independent stimuli (*A* and *B*) *via* two electromagnetic microvalves. In the resting state, the stimulus reservoirs are at atmospheric pressure and the solutions are continuously pumped through a V-shaped channel to an underpressurized waste container. Switching a valve (injection state) overpressurizes one reservoir, triggering the instant release of the stimulus solution through the injection channel (Fig. 1b,c). Both stimuli share the same delivery outlet, thus guaranteeing that no spatial clue is associated with switching between stimuli. The device can be positioned under a two-photon microscope for simultaneous recording of neuronal activity (Fig. 1a), while a fast camera monitors the larva’s tail movements from below, taking advantage of the chip’s transparency.

A crucial feature of this design is the negative pressure imposed on the waste container, which drives a co-flow of the stimulus solution and the chamber fluid in the downstream arm of the V-shaped channel. Although the flow rate is relatively low (of the order of 1*μL.s*^−1^), it prevents any cross-pollution between the buffer and the stimulus solution in the resting state, while guaranteeing through constant renewal that the stimulus solution at the entrance of the injection channels (i.e. ≈ 600 μm from the targeted sensory receptors) remains at the desired concentration.

### Flow kinematics of the microfluidic device

**Figure 2:**
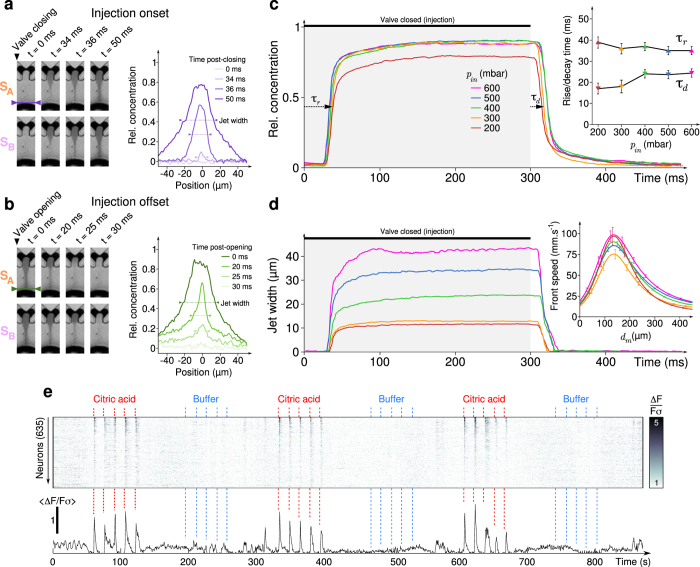
Flow characterization in the microfluidic device. (**a–b**) Left: kinematic view of the chamber during injection onset (**a**) and offset (**b**), for stimuli *S*_*A*_ and *S*_*B*_, recorded with a high-speed camera. For (**a**–**d**), *S*_*A*_ and *S*_*B*_ are an IR-dye used to characterize the flow dynamics. The region of interest is highlighted on Fig. 1b: the outlet of the delivery channel is at the top while the larva’s head tip is at the bottom of each frame. Right: Profiles of the relative concentration along a cross section of the chamber close to the larva’s mouth (purple and green lines on the kinematic view) at different moments after closing (**a**) or opening (**b**) the micro-valves. Jet’s width is defined as the width of the profile at half-maximum relative concentration. (**c**) Evolution of the relative concentration of the profiles shown in (a-b) for different reservoir pressures *p*_*in*_, averaged over 5 trials. Inset: rising (▲ *τ*_*r*_) and decay (▼ *τ*_*d*_) times such that the system reaches half the maximal relative concentration after microvalve switch, as a function of *p*_*in*_. (**d**) Evolution of jet width for different values of *p*_*in*_, averaged over 5 trials. Inset: Speed of the stimulus front during injection onset as a function of the distance to the larva’s mouth *d*_*m*_, for different *p*_*in*_. Same color code as in (**c**). (**e**) Raster (top) and average trace (bottom) of the normalized neuronal activity Δ*F*/*Fσ* in the vagal lobe during alternate presentations of citric acid (CA, first stimulus *S*_*A*_) and buffer (second stimulus *S*_*B*_) at *p*_*in*_ = 500 *mbar*. Neurons are ordered by decreasing responses to CA pulses. Similar responses were obtained for *n* = 10 larvae. For each channel, three trains of five 300 *ms*-pulses separated by 15 *s* were delivered. Error bars: standard deviations.

To characterize the performances of the microfluidic device we monitored the delivery of a dye-containing solution using high-speed videography. For this purpose, we switched one of the microvalves to instantly increase the pressure to *p*_*in*_ in one of the stimuli reservoirs. The time-evolution of the relative concentration, probed along a line tangential to the animal’s mouth (Fig. 2a,b), exhibited a rapid transition from zero (no dye) to one (nominal dye concentration) within a few tens of milliseconds (Fig. 2c). This transition dynamics was found to be highly reproducible and showed no significant dependence on the imposed pressure *p*_*in*_ for *p*_*in*_ ≥300 *mbar*. For each value of *p*_*in*_, we computed the onset and offset time-delays (noted *τ*_*r*_ and *τ*_*d*_, respectively) defined as the delays between the valve switching and the time at which the relative concentration reached half the maximal concentration (inset of Fig. 2c). We computed the evolution of the jet width (Fig. 2d), which showed a similar dynamics towards a plateau but with a plateau value that increased quasi-linearly with *p*_*in*_. It is worth noting that despite the jet is one order of magnitude thiner than the mouth’s size, it splits in two branches close to the head surface and flows symmetrically along the exposed parts of the head. The real surface of contact is thus determined by the amount of agarose removed around the larva’s mouth. Tracking the position of the dye/water interface during injection onset, we also estimated the velocity of the propagation front as a function of the distance to the mouth, for various values of *p*_*in*_ (inset of Fig. 2d).

These hydrodynamic recordings revealed several important features of the device. First, the stimulation onset and offset delays were small compared to typical gustatory stimulation times and were weakly dependent on injection overpressure. The valve switching sequence consistently drove the effective stimulus presentation sequence, albeit with a constant time delay. This device thus allows for near-millisecond precision on the stimulus presentation time, *i.e.* when the stimulus actually contacts the animal. The shortest stimulus duration that can be delivered by the device was ≈35 *ms*, as it corresponds to the time needed for the injection flows to reach a stationary state. We could also generate reproducible pulses of less than 10 *ms*, but in this regime the maximal concentration did not reach the nominal concentration. Second, the flow velocity on the fish face was independent of the injection pressure: it was imposed by the continuous buffer flow that pinches the stimulus jet and drags it to the animal. This process guarantees that the hydromechanical stress imposed on the larva is strictly invariant during and between stimuli presentations, with no modification at pulse onset and offset, such that no artifactual mechanosensory clues should accompany the chemical stimulation.

### Gustatory neuronal responses

To test whether the gustatory-stimulus delivery could also stimulate mechanosensory receptors, we examined gustatory-evoked neuronal responses elicited by pulse-like exposure to distinct flavours. We probed in particular the specificity of the neuronal response to gustatory inputs and the absence of associated activation of mechanosensory receptors around the larva’s mouth induced by change in the flow pattern. Two-photon calcium imaging was perfomed on six-to-seven day old Huc:GCaMP3 transgenic larvae^2^ first restrained in low-melting agarose (see **Sample preparation** in **Methods**). Prior to positioning them within the stimulation device, we carefully removed the agarose around the larva’s mouth in order to expose the lips and mouth cavity, a region rich in taste buds^11^,^12^,^13^.

The two stimulation channels were first used to alternatively deliver series of five 300 *ms*-long pulses of sour (citric acid, 10 *mM*) or tasteless (buffer, control) stimuli. The most responsive primary gustatory centers in teleost fish are the facial (VII), glossopharyngeal (IX) and vagal (X) lobes^14^. At the developmental stage for which the recordings were made the glossopharyngeal lobe is not yet fully-developed and is rather difficult to identify. In large-field imaging experiments (not shown) we could observe some activity in the vagal and facial lobes with acute citric acid stimulations, which suggests that most probably the stimuli activated both external (lips) and intraoral taste buds. The strongest responses where evoked in specific neuronal populations of the vagal lobe (Fig. 2e). Importantly, the control solution elicited no measurable activity, thus indicating that the measured evoked activity in the vagal lobe is solely representative of gustatory inputs rather than other variables associated with the stimulus (e.g. hydromechanical cues)

### Neuronal representation of gustatory stimuli with different hedonic values

**Figure 3:**
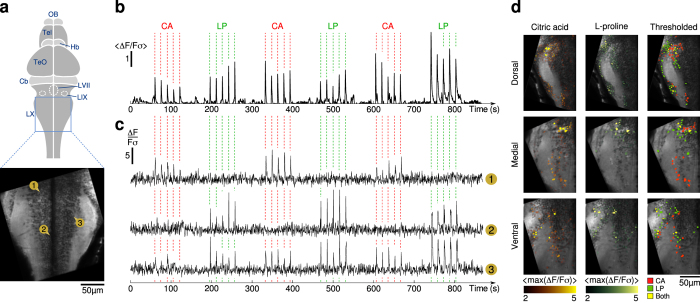
Neuronal responses to chemical stimulation. (**a**) Scheme of the larval brain and average fluorescence image of the vagal lobe. Neurons ① to ③, whose traces are shown in (**c**), are pinpointed. (**b**) Trace of the normalized neuronal activity averaged across all identified neurons of the vagal lobe. For each tastant, three trains of five 300 *ms* pulses separated by 15 *s* were delivered. (**c**) Normalized neuronal activity Δ*F*/*Fσ* of three neurons of the vagal lobe during alternate presentations of CA (stimulus *S*_*A*_) and LP (stimulus *S*_*B*_). Neuron ① responds exclusively to CA, ② responds exclusively to LP and ③ responds to both. (**d**) (Left, Middle) Maximum of Δ*F*/*Fσ* measured in the first 3 *s* following stimulus onset, averaged over 15 identical pulses, overlaid on the imaged plane (gray). (Right) Neurons responding to CA only (red), LP only (green) or both (yellow). *p*_*in*_ = 500 *mbar*.

The possibility offered by the microfluidic device to rapidly alternate the presentation of two stimuli allowed us to compare between the gustatory-induced activity patterns associated with distinct gustants in a single experiment. We illustrated this capability using two stimuli of distinct hedonic values: sour aversive, citric acid (CA) and appetitive umami, L-proline (LP). We sequentially delivered series of 300 *ms*-long pulses of either compound while recording neuronal activity in the vagal lobe (Fig. 3) . The mean signal, averaged over all identified neurons of the vagal lobe, show important responses at the population level (Fig. 3b), while at the single-cell level neuronal responses to either or both tastants have been observed (Fig. 3c). Although the topology of the gustatory-induced activity patterns appeared intermingled, we observed for three different planes (dorsal, medial and ventral) that a large fraction of neurons responded exclusively to either CA or LP, while a few responded to both (see Fig. 3d).

In the zebrafish larva, ascending projections carrying taste-related signals reach diencephalon and telencephalic areas. We thus similarly monitored CA and LP-induced responses in the dorsal telencephalic area (Supp. Fig. 3), a region known to respond to gustatory stimuli^15^. Interestingly, we found that even in higher gustatory centers, both CA and LP-induced responses remained relatively segregated.

### Effect of stimulus duration on gustatory-induced neuronal responses and motor behavior

**Figure 4:**
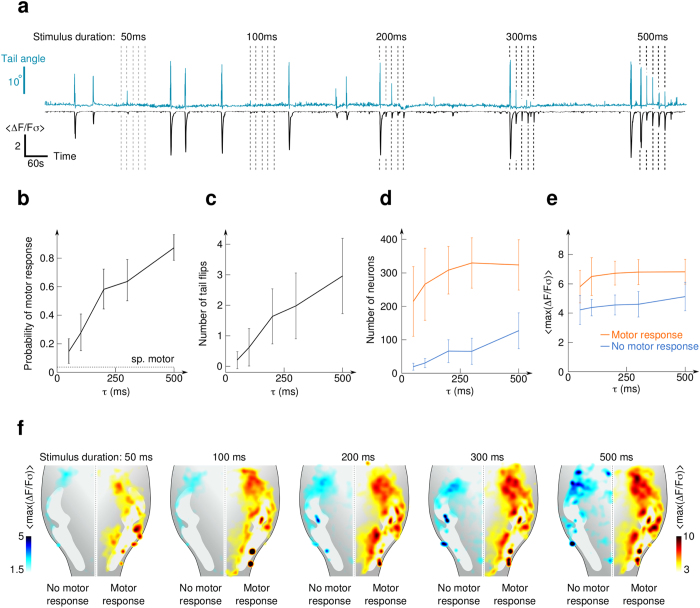
Effect of stimulus duration on behavioral and neuronal responses. (**a**) Time-traces of the tail angle and average normalized neuronal activity in the vagal lobe during trains of five similar stimuli of citric acid. The trains contained stimuli of increasing duration (50–500 *ms*). (**b**) Probability of observing at least one tail flip in a time interval of 3 *s* following the stimulus onset. The probability of a spontaneous tail movement in a random 3 *s* interval far from stimulation is close to zero (dashed line). (**c**) Average number of tail flips in a time interval of 3 *s* follwowing stimulus onset. (**d**) Number of neurons responding to the stimulation in trials for which no motor response (blue) or a motor response (orange) was observed. (**e**) Amplitude of the response (defined as the maximum of Δ*F*/*Fσ* in the 3 *s* post-stimulation) averaged across the responsive neurons in trials associated (orange) or not associated (blue) with a motor response. (**f**) Topographical organization of the gustatory-evoked neuronal response patterns in the vagal lobe circuit. The color code reflects the amplitude of the response averaged across trials where no motor response was evoked (left) and trials where a motor response was observed (right) for the five different stimuli durations. For (**b**–**f**), data were pooled from 11 larvae (22 half vagal lobes) to which 25 stimuli were presented as shown in (**a**). The recorded area is the same as in Fig. 3. Error bars: standard deviations. *p*_*in*_ = 500 *mbar*.

In aquatic species, the gustatory system is not only responsible for the decision of swallowing or rejecting ingested objects, but it also enables the animal to detect the location of potential food sources^6^ or to trigger escape from dangerous chemicals and water conditions^7^. Large amplitude tail deflections were robustly evoked by brief exposures to CA, an aversive tastant for the larva (Supp. Movie. 3 and 4). We took advantage of the unique time-precision of the microfluidic device and its suitability for simultaneous two-photon imaging and motor behavior video-monitoring to investigate the neuronal mechanisms underlying gustatory-induced motor behaviors (Fig. 4a).

We observed that acute CA stimulation induced discrete, one-sided tail flips (see Supp. Fig. 4c, Supp. Movie. 3 and Supp. Movie. 4) sometimes followed by short series of weaker oscillations, a behavior that is reminiscent of escape responses (C-turn). These responses occured with a probability that consistently increased with the stimulus duration (Fig. 4b). The average number of tail flips immediately following the stimulus presentation were also positively correlated with the stimulus duration (Fig. 4c). We found that the overall neuronal evoked response in the vagal lobe was larger when this was associated with a tail motor behavior. Thus, to probe the neural activity directly evoked by the gustatory inputs we separately analyzed the events that did not induce a tail flip.

For trials not associated with a motor response, we observed a quasi-linear relationship between the number of activated neurons and the duration of the chemical pulses (Fig. 4d, blue). Interestingly, the *Ca*^2+^ transient amplitude of the responding neurons were much less affected by the stimulus duration (Fig. 4e). When the stimuli induced a tail flip, an intense and extended neuronal response was measured as shown in Fig. 4d,f. The differences in topography for gustatory-induced responses associated or not associated with motor behaviors suggest that rostro-lateral regions are associated with the sensory response while more caudo-medial ones are associated with the gustatory-induced motor response, as illustrated in Fig. 4f.

These results demonstrate that the stimulus duration is mainly encoded by the number of activated neurons in specific regions of the vagal lobe. The latter drives in a probabilistic manner a discrete transition towards a neuronal-circuit state capable of inducing a tail-flip response.

### Olfactory-induced neuronal responses in the olfactory bulb

As a proof of concept, we performed experiments to validate the suitability of the microfluidic device for olfactory stimulation in zebrafish larvae. For this experiment, we presented to the larva’s nostril two odor-specific aminoacids (lysine and phenylalanine, 300 *ms* pulses, 10 *mM*) while monitoring changes in calcium dynamics for three different focal planes of the olfactory bulb along the dorso-ventral axis (dorsal, medial and ventral). We observed that the two odorants induced distinct neuronal response patterns (Supp. Fig. 4) as previously reported^16^.

## Discussion

In recent years, advances in soft-microlithography techniques have led to the design of microfluidic chips for the manipulation of small organisms^17^. Recently, a microfluidic high-throughput system has been proposed to simultaneously immobilize and orient tens of larvae while enabling functional imaging^18^. The miniaturization allows for fast modifications of the flow pattern to which the specimen is exposed while still operating in the laminar flow regime and mitigate cross-diffusion between the different solutions. Microfluidic approaches were also used for chemical stimulation in *C. Elegans*^19^,^20^.

Here, we designed an open-ended microfluidic chip that combines the advantage of miniaturization for precise control over fluid flow with the possibility to perform simultaneous behavioral and neuronal activity recordings on agarose-restrained zebrafish larvae. The micro-milling method enables direct micro- and macro-patterning on a single chip, such that the microfluidic channels (in the 100 *μm* range) could be directed onto an open pool (in the *cm* range) where the partially-restrained larva can be precisely positioned. This relatively simple fabrication method presents further advantages compared to standard soft-microlithography approaches: the circuit can sustain high pressures (in the *MPa* range), the channels dimensions can be adjusted in the 3 dimensions on 4 orders of magnitude with virtually no constraint on aspect ratio. These characteristics allow for precise adjustment of hydraulic resistances and flow pattern across the circuit. Furthermore, the chip can be rapidly replaced without changing the remote macro-scale delivery system (tubing, valves, pumps, stimuli reservoirs, etc.) through the use of custom-made connectors.

The capability of this chip to sequentially deliver multiple step-pulse-like stimuli, combined with whole-brain calcium imaging, opens new avenues to dissect the neuronal circuits underlying chemosensory processing in the vertebrate brain. First, it allows for the identification of tastants-specific neuronal networks across several trials and animals. This approach, which is routinely used in visual studies, was here implemented to identify neuronal circuits engaged in processing aversive versus appetitive information along the gustatory pathways.

The device unique capacity of delivering short square-pulse-like stimuli enabled us to study the neuronal mechanisms underlying gustatory-induced motor behaviors. We observed that the duration of the exposure to citric acid in the 50–500 *ms* range is mainly encoded by the number of recruited neurons in specific regions of the primary gustatory center, which in turn predicts both the probability of triggering an escape-like behavior and the number of induced tail movements. We thus suggest that gustatory perception in the zebrafish larva, *e.g.* the neuron-computational process leading to an adequate motor behaviour, takes place early in the gustatory pathway and is dictated by the number of neurons activated by the gustant.

The ability to rapidly switch between two distinct stimuli could be used in a straightforward way to study the neural processing involved in more complex stimulation sequences, and to analyse for instance how gustatory and olfactory stimuli interferes when presented with short time-lags.

Although we here focused on gustatory and olfactory neuronal processing *in vivo*, the same device can be directly used to probe the effect of transient exposure to drugs in *ex vivo* preparations. Given the large available library of zebrafish models of human syndromes, this technique has the potential to become a powerful tool for high-throughput vertebrate pharmaceutical screenings towards the development of new drugs.

## Methods

### Microfluidic chip fabrication

The chip design has been iteratively optimized to reduce the transition times (onset and offset of delivery). The angle at which the continuous circulating buffer flow is directed in the chamber has been determined with the use of a finite-element software (Comsol). For fabrication, we used a computer-controlled micromilling machine (Minitech Mini-Mill/GX) which allows for submicronic precision, and a custom set of programs to generate G-code machine instructions. A microscope (0.75× to 3× Microscope Body, Edmunds Optics) and camera where mounted on the micromilling machine to allow for precise positioning (≈1 *μm* in the tool axis direction) during tool change. Fabrication workflow was as follows: on a horizontal 40 × 40 × 4 *mm* PMMA slab, (*i*) the seven input/output through holes (600 *μm*-diameter drill, KT-0236-R, PMT Microtools, USA) were drilled, (*ii*) an area of 25 × 15 *mm* was surfaced with a 3 *mm* smoothing tool (3 *mm*-diameter radius-end mill, MSRS 230, NS Tools, Japan) to obtain a flat reference surface (optical grade), (*iii*) channels were milled (100 *μm*-diameter mill, TR-2-0040-S, PMT Microtools, USA), and finally (*iv*) the open chamber was milled (500 *μm* and 3 *mm*-diameter mills, resp. TR-2-0200-S and SR-2-1181-S, PMT microtools, USA). The cover was milled out of a 22 × 27 × 0.25 *mm* thin PMMA sheet (ME303005, Goodfellow) using a 500 *μm*-diameter mill (TR-2-0200-S, PMT microtools, USA) to match the shape of the chamber such that the latter remains open after sealing. The chip and cover were washed three times for 5 min in 25% isopropanol and manually aligned under a microscope. Short laser pulses (45 *W*, Hobby 5^*th*^ gen., Full Spectrum Laser, USA) were used to locally melt the PMMA at distant locations from the channels in order to secure alignment during chemically-assisted thermal bonding. Some chips used in this work were sealed with a DMSO/methanol-based melting agent, following the protocol of Ref. 21, but our most recent chips were sealed with a custom protocol that provided better resistance with a less toxic melting agent: 50% acetic acid at 65°C for 15 min under a 1.7 *MPa* load per chip. The chips were rinsed several times with distilled water before use.

### Flow control and measurements

A custom brass connector (see Supp. Fig. 1-b) was screwed onto the lower side of the chip to connect the input/output channels to the pressure control manifold, the push-pull syringe pump and the waste container. The continuous buffer flow in the chamber was generated by a push-pull syringe (Legato 270, Kd Scientific), operated at a constant volumic flow rate of 2*μL.s*^−1^. A custom-made duralumin manifold was used to connect the chip, the stimulus reservoirs and the microvalves. Two 3-way electro-microvalves (LHDA0533115H, The Lee Company) controlled the pressure in the stimuli reservoirs. An I/O device (Arduino UNO) was used to send TTL pulses to an amplifier connected to the microvalves and to trigger the high-speed camera. Microvalves were normaly opened on atmospheric air *p*_0_ and closed on a pressure source *p*_*in*_, most generally set at 500 *mbar* overpressure. The waste reservoir surface was positioned below the chamber, which set its pressure ≈ −50 *mbar*. Flow recordings were performed with a high-speed camera (Fastcam APX-RS, Photron, Japan) operated at 1 *kHz* under infra-red light (850 *nm*, SFH4750, Osram). An infra-red dye (IR-806, Sigma) was dissolved in distilled water (*c*_0_ = 0.34 *mM*). Traces of precipitate were eliminated *via* filtering (0.22 *μM*, Millipore). To obtain concentrations from pixel intensities, we measured the molar absorptivity of the dye solution (*ε* = 600 *m*^2^*.mol*^−1^) and applied Beer-Lambert’s law: *c* = *log*_10_(*I*_0_/*I*)/*εl*, where *I*_0_ and *I* are the pixel intensities with buffer and dye and *l* is the thickness of the channel. Relative concentrations were computed as *c*/*c*_0_. Tastant and odorant stock solutions (Sigma) were made at 100 *mM* in distilled water and diluted in embryo medium just before imaging to reach the concentration of 10 *mM*.

### Zebrafish larvae

Transgenic zebrafish larva expressing a genetically encoded calcium indicator GCaMP3 under a pan-neuronal promoter, HuC (as described in Ref. 2) was used for all experiments. The HuC:GCaMP3GS5 line embryos were collected and raised at 28.5 °C in E3 embryo medium. Larvae were kept under 14/10 hours on/off light cycles and fed after 6 d.p.f. All experiments were carried out in accordance with approved guidelines and approved by *Le Comité d'Éthique pour l’Expérimentation Animale Charles Darwin* (Ce5/2009/027).

### Retrograde Labeling

To label the reticulospinal and vestibulospinal neurons in the hindbrain, we performed retrograde labeling by injecting a Dextran Texas Red dye (10,000 MW, Invitrogen) in the spinal cord. One day prior to imaging, 5 days-old HuC:GCaMP3 zebrafish larvae were embedded in 2% low-gelling agarose. The larva was placed on their side in a drop of Ringer solution. A 50% solution of Dextran Texas Red in 10% Ringer solution was pressure-injected *via* a glass microelectrode (10–20 *μm* tip diameter) inserted into caudal spinal cord at the level of the anus using 3 × 100 *ms* pulses. The dye penetrated the spinal cord axons *via* the damaged axons. After injection the fish were allowed to recover and kept in fish embryo media until further use. A day later, the larva was embedded in 2% low-gelling agarose. Fluorescent-labeled reticulospinal and vestibulospinal neurons were imaged using two-photon scanning microscopy. This experiment was used as a clear landmark for the recognition of the vagal lobe ≈30 *μm* dorso-caudally to the reticulospinal and vestibulospinal neurons (see Supp. Fig. 2).

### Sample preparation

Six to seven days old zebrafish larvae were embedded in a drop of low-gelling agarose (2%, Sigma) on a movable slider (see Supp. Fig. 1-d). The slider was placed into an acrylic mold whose shape exactly corresponds to the pool without the channels. The larva was plunged into liquid agar at ≈30°C and a drop containing the larva was placed on the slider. The position of the larva could be slightly modified during the gelification process by moving surrounding agar with surgical tools. After some practice, we could optimize the position of the larva such that the head was very close to the slider tip and the body axis in the direction of the delivery channel. The slider was designed with an array of tiny holes (100 *μm* in diameter) at the location of the specimen to provide a strong grip between the slider and agar. Once agar had gelified, we removed the slider and gently cleared the larva’s face with a dissecting pin under a binocular to expose its upper and lower lips (gustatory) or nostrils (olfactory). For experiments in which behavior was recorded, the tail tip was also cleared. For imaging experiments in which we did not monitored behavior, the larva was paralyzed in 300 *μM* pancuronium bromide (Tocris) added directly to the agarose. The preparation was kept in embryo medium until the slider was transferred to the microfluidic chip. A bath of embryo medium was maintained on top of the chip throughout the experiments. During slider insertion, it was first introduced into the bath and then positioned in the pool to avoid air bubbles. All experiments were performed in the dark.

### Two-photon calcium imaging

The two-photon microscope was based on a MOM system (Sutter) with a 25× objective (NA1.05, Olympus) and a laser tuned at 920 *nm* (Mai Tai DeepSee Ti:Sapphire). The output power at the focal plane was less than 3 *mW*. The filters consisted of an objective dichroic (FF705), a short-pass (IR Blocker, AFF01-680) and a band-pass filter (FF01 520/70), all from Semrock. The PMT was a H1070 (GaAsP) from Hamamatsu. The emission signal was pre-amplified with a SR-570 (Stanford Research Systems) and acquired using ScanImage at 1.95 *Hz*, with 256 × 256 pixels resolution. Recordings were made at three different optical sections, 20 *μm* apart. To maintain consistency between different recordings and in different animals, each layer was identified based on several anatomical landmarks (*e.g.* cell density, neuropil shape and size).

### Image Processing

Calcium imaging videos were first registered using the Turboreg plugin for ImageJ. Then, regions of interest (ROIs) corresponding to every morphologically identifiable neuron were semi-automatically defined using a custom program. In GCaMP strains, fluorescence is mainly localized in the cytosol with minimum penetration to the cellular nuclei, so we first identified individual nuclei as local intensity minima. To obtain neuronal contours we computed the Euclidean distance of each pixel to the nearest minima and performed a watershed segmentation. ROIs typically included neuronal nuclei and a thin cytosolic surrounding ring, excluding the outermost cytosolic perimeter that could potentially be subject of cross-neuron fluorescence contamination due to high neuronal density. The obtained ROIs were manually inspected and corrected when necessary. Eventually, the fluorescence traces were computed as the average intensity over each ROI. To record motor behavior, a miniature microscope connected to a fast infrared camera (120 *Hz*, TXG02, Baumer) was placed below the microfluidic device. We computed the raw orientation of the binarized image of the tail, and tail angle was defined as the deviation from the baseline orientation^22^,^23^. The tail beats after each pulse were counted by eye. All algorithms were written in Matlab.

### Neuronal data analysis

A baseline fluorescence signal was estimated for each neuron by computing the 8^*th*^ percentile of fluorescence traces *F*(*t*) in sliding windows of 15 *s*^24^. The resulting smooth curve *b*(*t*) locally approximated the baseline level and reflected slow fluctuations unrelated to the fast calcium transients evoked by neuronal activity. The relative variation of fluorescence intensity was calculated as Δ*F*/*F* = (*F* − *b*)/*b*. In a typical experiment, trains of stimulations separated by 15 *s* alternate with phases without stimulation. We defined the resting times as the set of times for which no stimulation was delivered in the previous 15 *s*. For each neuron, we computed the standard deviation of the relative variation of fluorescence during resting times *σ* = *std*(Δ*F*/*F*)_*rest*_ and the normalized relative variation of fluorescence intensity Δ*F*/*Fσ*. To isolate neurons responding to a stimulation pulse, we computed *m* the maximum of Δ*F*/*Fσ* in the first 3 seconds post-stimulation (seven images). To determine which neurons were significantly responding, we performed a Kolmogorov-Smirnov test to determine whether each value of *m* could arise from the distribution of the maximum of a set of seven normally distributed random points. A maximum of *m* = 3.189 corresponds to a p-value of 0.01, and was chosen as the responding threshold. For each fish, both sides of the time-averaged image of the vagal lobe were semi-manually registered onto a virtual “average” half-vagal lobe. Average patterns of activity were then obtained by convoluting the discrete field 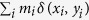 of neurons stemming from several animals (11 larvae, hence 22 half-vagal lobes) with a Gaussian kernel of width *σ*_*k*_ = 4.2 *μm* and unit height. These maps were normalized by the density map, computed as the convolution of 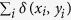 with a Gaussian kernel of width 2*σ*_*k*_ and height 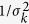.

## Additional Information

**How to cite this article**: Candelier, R. *et al.* A microfluidic device to study neuronal and motor responses to acute chemical stimuli in zebrafish. *Sci. Rep.* **5**, 12196; doi: 10.1038/srep12196 (2015).

## Supplementary Material

Supplementary Movie 1

Supplementary Movie 2

Supplementary Movie 3

Supplementary Movie 4

Supplementary Information
