# Supplementary material for: A microfluidic device to study neuronal and motor responses to acute chemical stimuli in zebrafish
Source: Sci Rep. 2015 Jul 21;5:12196. doi: 10.1038/srep12196 (PMC4508848; doi:10.1038/srep12196)
Supplement: Supplementary Information [file srep12196-s5.pdf]

A microfluidic device to study neuronal and motor responses to acute chemical stimuli in zebrafish.

Raphaël Candelier[1,2,†], Meena Sriti Murmu[3,4,5,†], Sebastián Alejo Romano[3,4,5], Adrien Jouary[3,4,5], Georges Debrégeas[1,2,‡] and Germán Sumbre[3,4,5,‡]

[1]Sorbonne Universités, UPMC Univ. Paris 06, UMR 8237, Laboratoire Jean Perrin, F-75005 Paris, France

[2]CNRS UMR 8237, Laboratoire Jean Perrin, F-75005 Paris, France

[3]Ecole Normale Supérieure, Institut de Biologie de l'ENS, IBENS, Paris, France

[4]INSERM, U1024, 75005 Paris, France

[5]CNRS, UMR 8197, 75005 Paris, France

May 28, 2015

Movie captions

Movie 1

Delivery of a single pulse of a dye-containing solution, recorded at $1\,kHz$. The valve (not visible) opens for $100\,ms$ and the solution contacts the fish face for $85\,ms$.

Movie 2

Delivery of multiple $50\,ms$-long pulses of two dye-containing solutions, recorded at $500\,Hz$. The two microvalves are activated alternatively to trigger sequential injections from both channels. Channel 1 opens for $50\,ms$ at $t = 100$, 500 and $900\,ms$, channel 2 opens for $50\,ms$ at $t = 300$, 700 and $1100\,ms$. Solutions contact the fish face for $\approx 25\,ms$.

Movie 3

Behavioral response to $300\,ms$-long pulses of citric acid, recorded at $50\,Hz$. The tail of the larva is freed from agar, and its absolute angular trace is displayed in blue on the bottom-right plot. Five stimuli, indicated by the red rectangles, are delivered at $t = 5$, 10, 15, 20 and $25\,s$. Robust behavioral responses appear approximatively $500\,ms$ after each stimulus onset. Spontaneous bursts of behavioral activity sporadically occur.

Movie 4

Simultaneous recordings of neuronal and behavioral responses to $300\,ms$-long pulses of citric acid. The traces of the normalized neuronal activity $\Delta F/F\sigma$ averaged over all neurons (yellow) and the tail angle (magenta) are displayed on the bottom plot. Five pulses-like stimuli, indicated by grey rectangles, are delivered at $t = 18$, 33, 48, 63 and $78\,s$.

Supplementary Figures

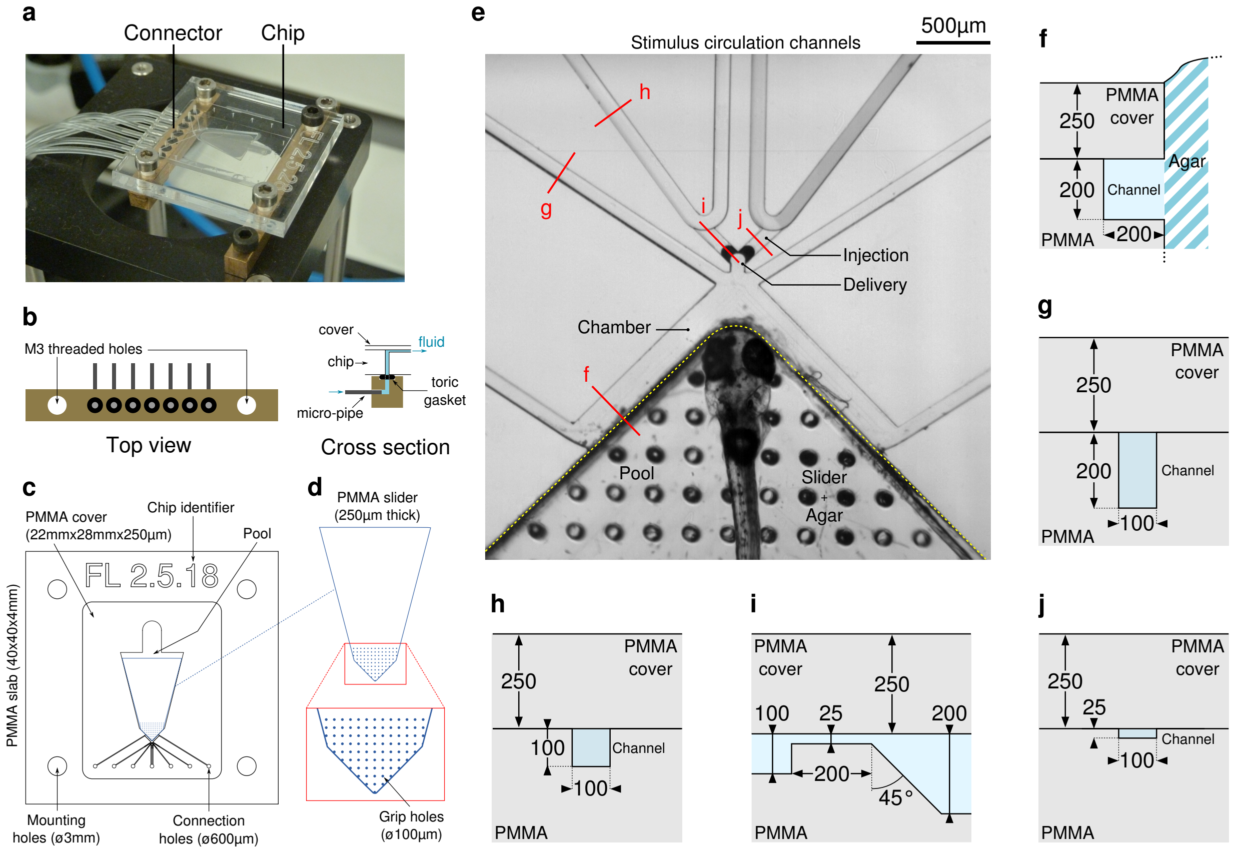

Supplementary Figure 1. Detailed description of the microfluidic chip. (**a**) Picture of the chip mounted on the brass connector (left) and the brass holder (right). (**b**) Top view and cross section schemes of the connector. The brass part is $40 \times 5 \times 5\, mm$ and micro-pipes have an external diameter of $600\, \mu m$. Toric gaskets prevent leakage when the chip is screwed onto the connector. (**c**) Scheme of the chip. It is made of a micro-milled PMMA slab sealed with a thin PMMA cover. (**d**) Scheme of the larva-holding slider. The blow-up shows the grip holes at the slider tip, which ensure a strong binding of the agar drop. (**e**) Microfluidic channels around the larva's head. The dashed yellow line indicates the separation between the sealed part (covered) and the pool (not-covered), in which the slider holding the agaros-embedded larva is inserted. Red segments indicate locations of the cross-sections displayed in panels (f-j). (**f-j**) Cross section schemes of the different channels. In (f) one wall of the chamber channel is made of gelified agarose. It is replaced by the larva's face at the slider tip. The 45° slope in (i), visible as a dark spot in (e), prevents the formation of vortices and stagnant volumes in the delivery channel.

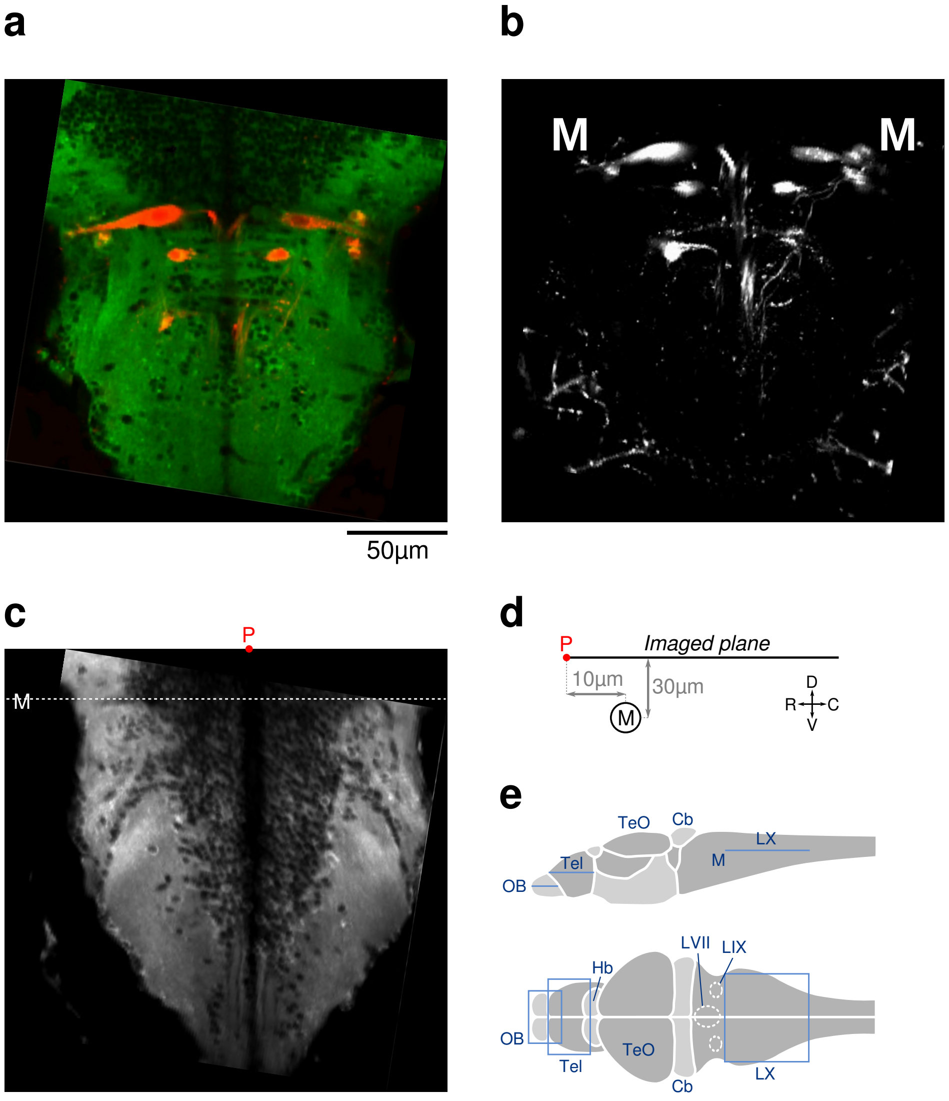

Supplementary Figure 2. Localization of the recorded brain regions. (**a, b**) Fluorescence images of reticulospinal neurons retrogradely labeled with Dextran Texas-red. Mauthner cells (M) are clearly recognized. (**c**) Image of the vagal lobe obtained by two-photon microscopy. Recordings were made in areas that lied $\approx 30\,\mu m$ dorsally with respect to the reticulospinal and the vestibulospinal neurons, whose approximative dorso-ventral location is represented with a dashed line. (**d**) Scheme of the position of the imaged plane with respect to Mauthner cells (sagittal view). The virtual point P is also represented in panel c for correspondence. (**e**) Schemes of the larva's brain and areas where neuronal recordings were performed (blue rectangles). OB = olfactory bulb, Tel = telencephalic lobes, Hb = habenula, TeO = optic tectum, Cb = cerebellum, LVII = facial lobe, LIX = glossopharyngeal lobe, LX = vagal lobe, M = Mauthner cell.

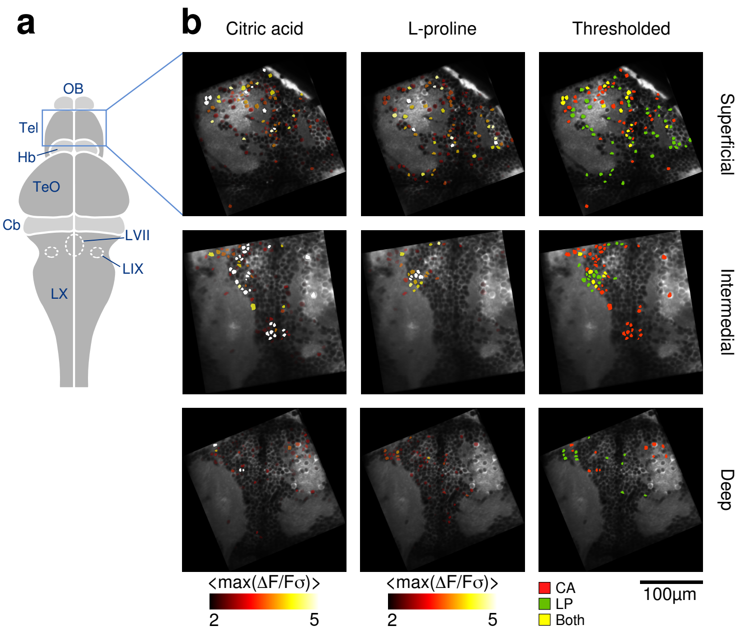

Supplementary Figure 3. Gustatory responses in the superficial, intermedial and deep layers of the dorsal telencephalon evoked by 300 ms-pulses of citric acid (CA) and L-proline (LP). The three focal planes were imaged 20µm apart, thus a total depth of 40µm. (**a**) Scheme of the larva brain. The recorded region is highlighted by a blue rectangle. (**b**) *Left, Middle* Maximal value of $\Delta F/F\sigma$ measured in the first 3 s post-stimulation, averaged over $n = 15$ presentations, overlaid on the time-average fluorescence signal (grey image). *Right* Neurons responding to either CA only (red), LP only (green) or both (yellow). $p_{in} = 500\,mbar$.

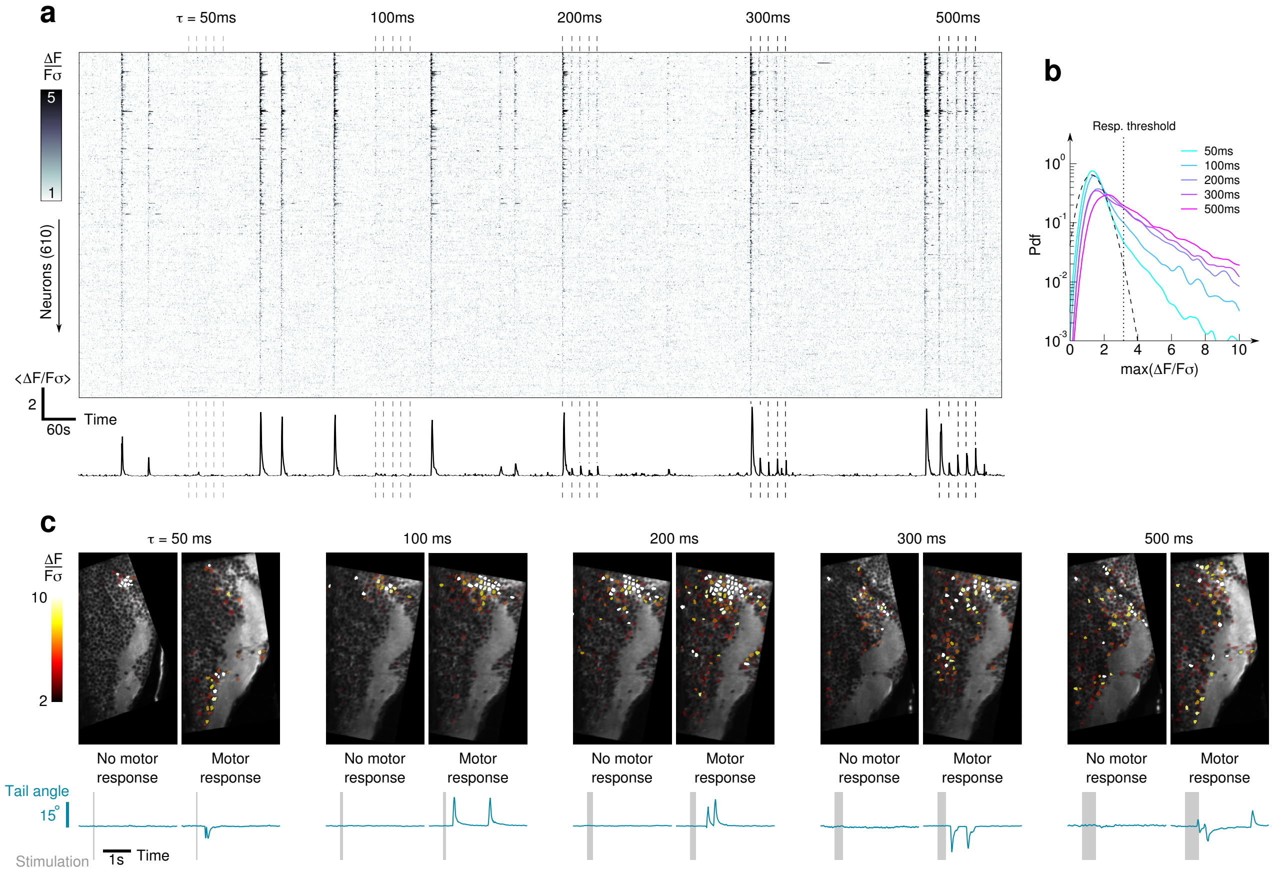

Supplementary Figure 4. Additional information on the effect of stimulus duration. (**a**) Raster plot (top) and $\Delta F/F\sigma$ averaged across neurons (bottom) for the same experiment as in **Fig. 4-a**. (**b**) Distributions of the maximal values of $\Delta F/F\sigma$ in the $3\,s$ post-stimulation for the different stimuli durations. The black dashed curve is the distribution of maximum values of normally distributed random noise. The dotted vertical line indicates the responding threshold (3.189). (**c**) Exemples of neuronal and behavioral responses to citric acid stimuli.

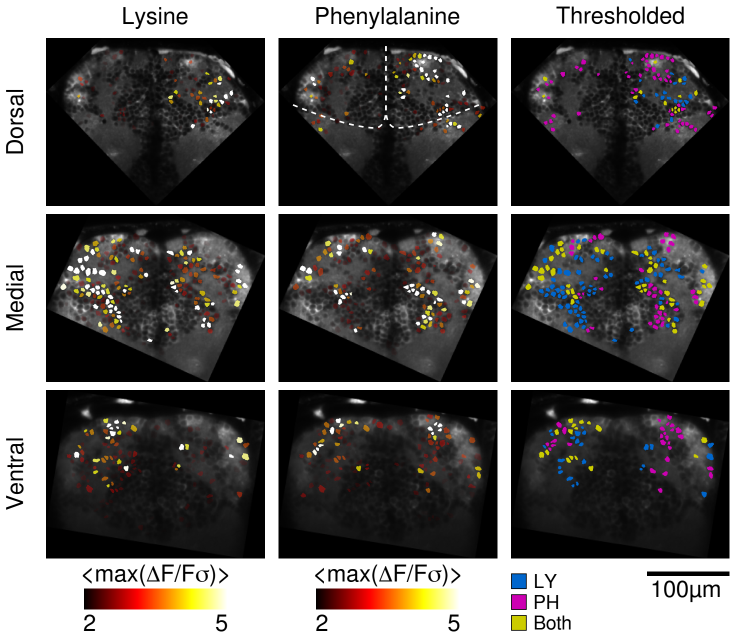

Supplementary Figure 5. Olfactory responses in the dorsal, medial and ventral regions of the olfactory bulb evoked by $300\,ms$-pulses of Lysine (LY) and Phenylalanine (PH). (**Left, Middle**) Maximal value of $\Delta F/F\sigma$ measured in the first $3\,s$ post-stimulation, averaged over $n = 15$ presentations, overlaid on the time-average fluorescence signal (gray image). The dashed white line indicate the boundary between the olfactory bulb and the telencephalic lobes. (**Bottom**) Neurons responding to either LY only (blue), PH only (purple) or both (yellow). $p_{in} = 500\,mbar$.

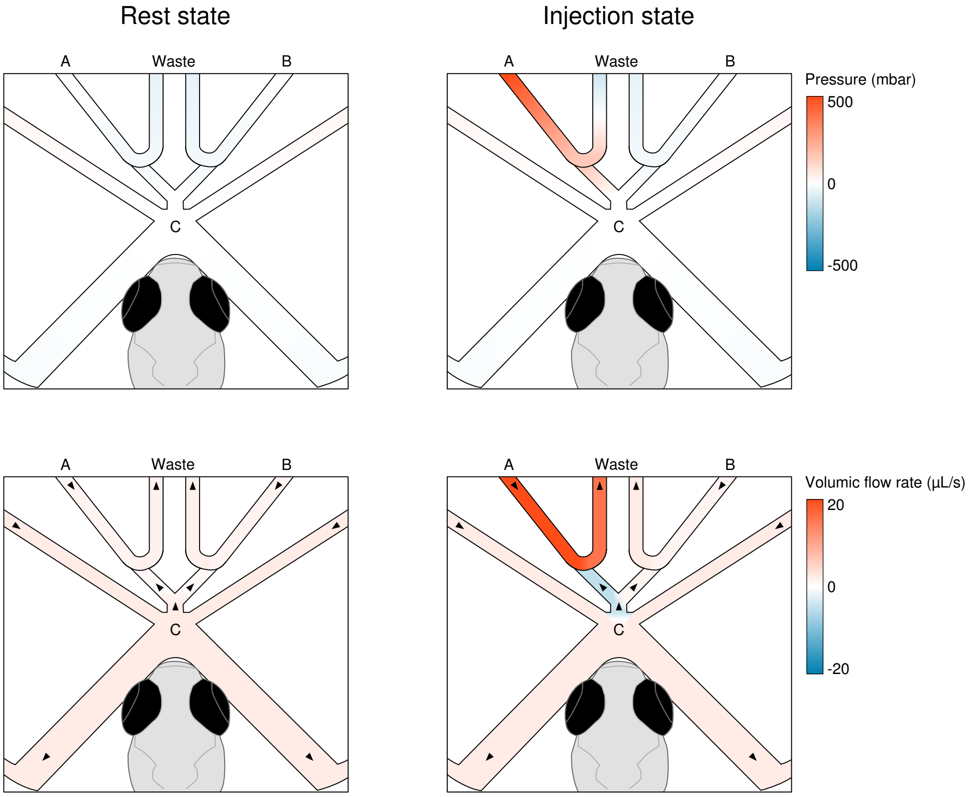

Supplementary Figure 6. Pressure (top) and flow rates (bottom) fields for the two stationnary states, at rest (left) and during injection (right), determined with eq. **1** and eq. **4**. The boundary conditions are $p_B = 0\,mbar$, $p_D = -0.05\,mbar$, $p_w = -50\,mbar$ and $p_A = 0\,mbar$ in the resting state. In the injection state, p_A is set to $500\,mbar$. In the bottom panel, small black arrows indicate the direction of positive flow rate in each channel, defined as the direction of flow rate in the resting state.

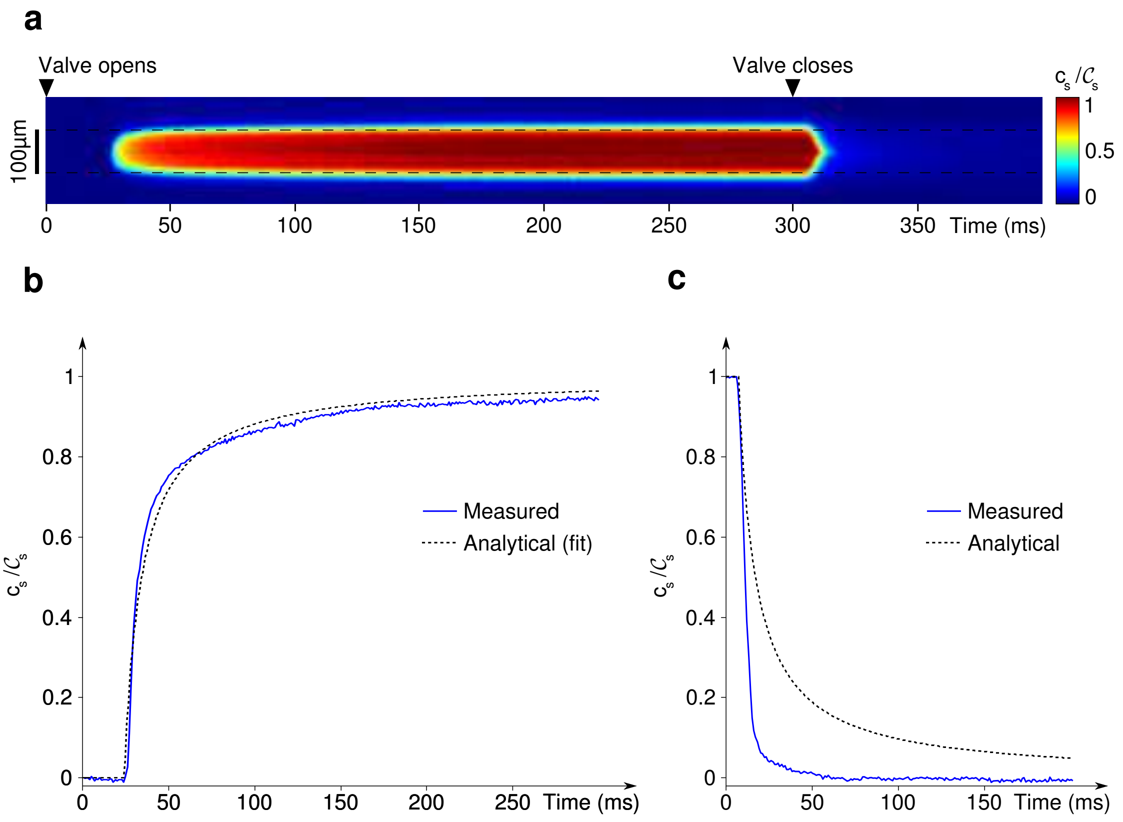

Supplementary Figure 7. (**a**) Evolution of the relative concentration profile close to the outlet of the delivery channel as a function of time, measured in the chamber of our microfluidic device. The position of the $100\,\mu m$ channel outlet is shown with black-dashed horizontal lines. (**b**) Evolution of the average relative concentration profile of stimulus $c_s/\mathcal{C}_s$ at the onset of stimulus delivery, close to the outlet of the delivery channel. The analytical curve is a fit corresponding to eq. **9b** with $\tau = 10.2\,ms$. (**c**) Evolution of the average relative concentration profile of stimulus $c_s/\mathcal{C}_s$ at the offset of stimulus delivery, close to the outlet of the delivery channel. The analytical curve corresponds to eq. **9a** with the same τ as in (b).

Supplementary information

Fluid dynamics of chemical delivering

Stationary flows

Our microfluidic system has two stationary states, namely resting and injection, depending on the pressure applied to the solution reservoirs. In this section, we derive analytical expressions for pressure and volumic flow rates in all channels for both states, which provides a complete description of the flows inside the chip.

The hydrodynamics in the chamber channels and the delivery circuit are rather decoupled, such that these circuits can be separated. The microfluidic delivery circuit can be synthesized by the following diagram, where P, q and R stand for pressure (all expressed as offsets to the atmospheric pressure), volumic flow rate and hydraulic resistance:

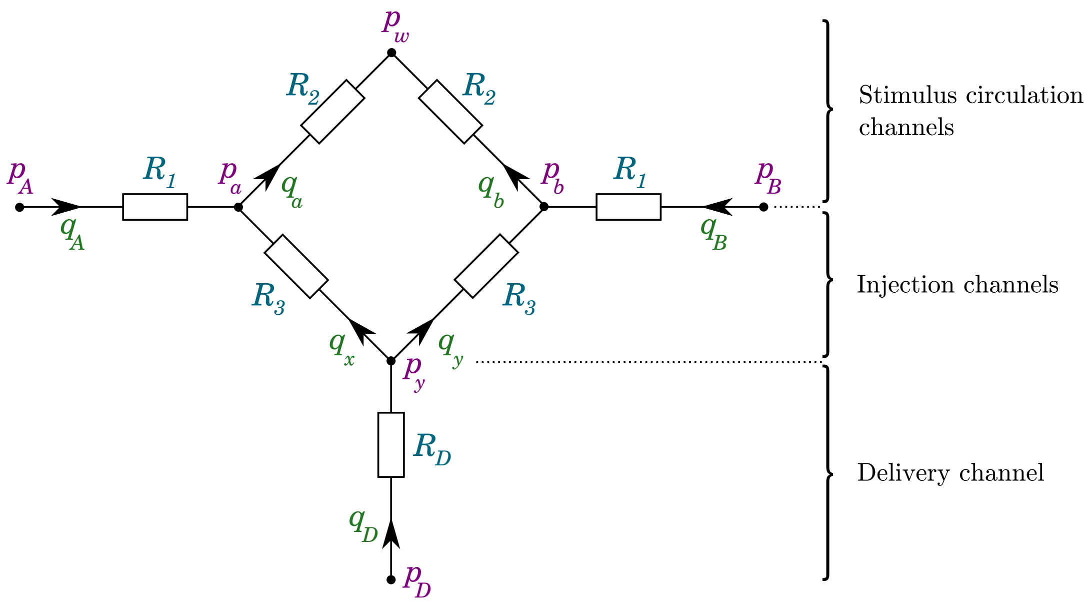

Supplementary diagram 1. Equivalent diagram of the delivery circuit. Pressures are defined at the nodes and each channel is described by its hydraulic resistance R and flow rate q. Arrows indicate the direction of positive flow rates, defined as the direction of fluid in the resting state.

The pressures of the stimuli reservoirs p_A and p_B and of the waste p_w are directly controlled by the operator, while the pressure at the end of the delivery channel p_D is very close to atmospheric pressure[1]. The pressures p_a, p_b and p_y are set internally. For each of the seven segments the Hagen–Poiseuille relation applies :

$$\Delta p = Rq \tag{1}$$

where the hydraulic resistance R of a rectangle channel of width w, height h and length L is defined by :

$$R = \frac{8\mu L}{\pi}\left(\frac{w+h}{wh}\right)^4 \tag{2}$$

with $\mu = 10^{-3} Pa.s$ the dynamic viscosity of water. In units of $10^8\, mbar.s.m^{-3}$, the hydraulic resistances are $R_1 = 170$, $R_2 = 132$, $R_3 = 320$ and $R_D = 1.3$.

We obtain three more equations with flow conservation:

$$q_A + q_x = q_a \tag{3a}$$

$$q_B + q_y = q_b \tag{3b}$$

$$q_D = q_x + q_y \tag{3c}$$

[1] p_D is controlled by the flow rate in the chamber such that $p_D = -\rho_w q_{ch}^2/2S_{ch}^2$, with ρ_w the volumic mass of water, $q_{ch} = 2\mu L.s^{-1}$ the flow rate in the chamber and $S_{ch} = 4.10^{-8} m^2$ the surface of the cross-section of the chamber channel, which gives $p_D = -0.05\, mbar$.

The system of ten linear equations in eq. 1 and eq. 3 can be solved. Introducing the reduced quantities $\alpha = \frac{R_3}{R_D}$, $\gamma_1 = \frac{R_2R_3}{R_1R_2+R_2R_3+R_1R_3}$, $\gamma_2 = \frac{R_1R_3}{R_1R_2+R_2R_3+R_1R_3}$ and $\gamma_3 = \frac{R_1R_2}{R_1R_2+R_2R_3+R_1R_3}$, the internal pressures write:

$$p_y = \frac{\alpha p_D + \gamma_1(p_A + p_B) + 2\gamma_2 p_w}{\alpha + 2(\gamma_1 + \gamma_2)} \quad (4a)$$

$$p_a = \gamma_1 p_A + \gamma_2 p_w + \gamma_3 p_y \quad (4b)$$

$$p_b = \gamma_1 p_B + \gamma_2 p_w + \gamma_3 p_y \quad (4c)$$

The pressures and flow rates in the chamber circuit can be calculated in a similar way. All pressures and flow rates are displayed on **Supplementary Figure 6**, in both the resting and injection states.

The flow rate at the outlet of the delivery channel q_D follows an affine dependence with the pressure applied in the stimulus reservoir : $q_D = a.p_A + b$, with $a = -1.10 \times 10^{-11}\, m^3.mbar^{-1}.s^{-1}$ and $b = 1.42 \times 10^{-9}\, m^3.s^{-1}$. At the typical pressure at which recordings have been performed ($p_A = 500\, mbar$) we found $q_D = -4.08\, \mu L.s^{-1}$. Conversely, the reservoir pressure at which the outlet flow becomes negative, *i.e.* the minimal pressure for which the system is injecting stimulus in the chamber, is at $p_A^{min} = 128\, mbar$. This is coherent with the minimal pressure found experimentally, namely $125\, mbar$.

Comparison with standard injection methods

In this section, we discuss how the dynamical aspects of our system compares with methods that have been most commonly used for delivering chemicals to aquatic species.

Methods with uncontrolled advection/diffusion

A class of methods is based on local concentration increase, either *via* a solid media (*e.g.* soaked cotton pellets [1, 2], freeze-dried pellets [3], starch gel[4], agar gel [5]) or by directly releasing drops of the product in the sample tank [6].

The transport of chemical compounds involves two distinct mechanisms, advection and diffusion [7]. In most cases, advection is a much faster transporting process, and diffusion can be neglected. In the previously cited methods, advection processes are not controlled (flows created in the fluid during product release, movements of the animal, thermal convection, etc.) which introduce a huge variability in the front propagation speed and important concentration heterogeneity. These methods are viable in studies where temporal aspects or control of the concentration are not important, but a precise understanding of the gustatory neuronal processes requires a better level of control. Hence, to generate sharp and reproducible chemical stimulations, it is crucial to control the advection processes around the specimen.

In addition, in advection-based delivery systems where the chemical is stored very close to the sample, care should be taken to eliminate diffusion-based cross-pollution. Indeed, diffusion should be avoided for two reasons: (i) since the propagation front spreads with time, the concentration at a given point slowly increases which makes it difficult to define an onset time of stimulation, and (ii) since diffusion is an irreversible process, without proper cleaning of the chemoreceptors the delivery cannot be repeated on the same animal, which hampers multi-trials averaging. Diffusion is a rather slow process[2], but can appear during the course of a experiment. In our microfluidic device, the distance between the stimuli circulation channels and the larva's mouth is $\approx 375\, \mu m$, so a front of stimulus could reach the sample in a few minutes. Our system has thus been designed such that in the resting state a permanent flow is maintained in the injection and delivery channels to suck water from the chamber (q_x, q_y and q_D are positive), which completely eliminates diffusion-based cross-pollution, while the flow around the sample is unchanged between the resting and injection states.

Valve-driven injection

Common advection-based methods involve a switching valve system (see *e.g.* [9, 10] for the gustatory system or [11, 12, 13] for the olfactory system). In these devices a continuous flow is set into an injection tube, and the fluid (buffer or stimulus) is switched at the inlet of the injection tube. The valve prevents cross-diffusion between the buffer and the stimulus solution and ensures a constant flow rate around the specimen. In this section, we compute the evolution of the concentration at the outlet of such an injection tube and show that it has a slow evolution, which we compares to measurements in our device.

Let us consider a cylindrical[3] tube of length L and internal radius ρ in which a continuous flow is imposed at a constant flow rate q (see **Supplementary diagram 2**). We also assume that at time $t = 0$, solution A

[2]For instance, citric acid has a diffusion coefficient of $D \simeq 0.65 \times 10^{-9}\, m^2.s^{-1}$ in water[8], which gives a propagation of the diffusion front in $\approx 15\, s$ for a distance of $100\, \mu m$, and $\approx 385\, s$ for a distance of $500\, \mu m$.

[3]Similar results are found with rectangular channels, though the calculus is more complicated. See [14] for details.

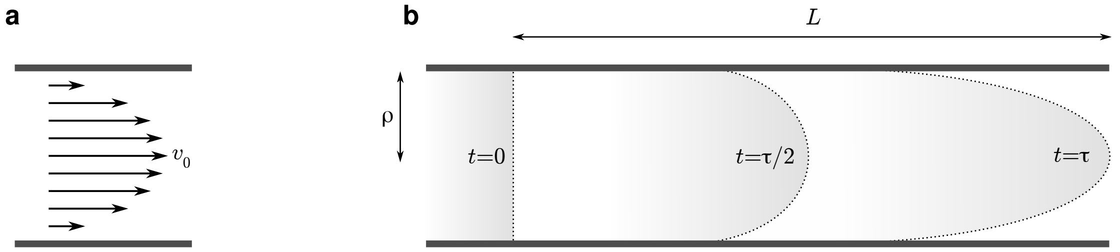

Supplementary diagram 2. (**a**) Velocity profile of a Poiseuille, laminar flow. (**b**) Scheme of the evolution of a planar front in an injection tube.

is switched to solution B using a perfect valve at the entrance of the tube, forming a planar front. The front deforms as solution B progresses in the tube with a Poiseuille flow profile :

$$v(r) = v_0 \left(1 - \left(\frac{r}{\rho}\right)^2\right) \tag{5}$$

where v_0 is the maximum velocity, at the center of the tube. With this expression the flow rate can be derived as a function of v_0 :

$$q = \int_0^\rho 2\pi r v(r)\, dr = 2\pi\rho^2 v_0 \int_0^1 (1-x)\, dx = \frac{\pi\rho^2 v_0}{2} \tag{6}$$

At $t = \tau$, the front tip reaches the outlet of the injection tube. This time is defined by :

$$\tau = \frac{L}{v_0} = \frac{\pi L \rho^2}{2q} \tag{7}$$

Let us note $\mathcal{C}_A$ and $\mathcal{C}_B$ the concentrations of solutions A and B. We aim at computing the time-evolution of the average concentrations $c_A(t)$ and $c_B(t)$ at the tube outlet :

- For $t < \tau$, we have : $c_A(t) = \mathcal{C}_A$ and $c_B(t) = 0$.
- For $t \geq \tau$, the concentrations are given by :

$$c_A(t) = \mathcal{C}_A \left(1 - \left(\frac{r_f(t)}{\rho}\right)^2\right) \tag{8a}$$

$$c_B(t) = \mathcal{C}_B \left(\frac{r_f(t)}{\rho}\right)^2 \tag{8b}$$

where $r_f(t)$ is the radius of the cross section of the front at the outlet of the tube. The radius $r_f(t)$ corresponds to a velocity of exactly $v(r_f(t)) = L/t$, which gives, by using the expression of the Poiseuille profile in eq. 5 :

$$\frac{L}{t} = v_0 \left(1 - \left(\frac{r_f}{\rho}\right)^2\right)$$

hence :

$$\frac{L}{t} = v_0 \frac{c_A(t)}{\mathcal{C}_A}$$

$$\frac{L}{t} = v_0 \left(1 - \frac{c_B(t)}{\mathcal{C}_B}\right)$$

and, finally :

$$c_A(t) = \mathcal{C}_A \frac{L}{v_0 t} = \mathcal{C}_A \frac{\tau}{t} \tag{9a}$$

$$c_B(t) = \mathcal{C}_B \left(1 - \frac{L}{v_0 t}\right) = \mathcal{C}_B \left(1 - \frac{\tau}{t}\right) \tag{9b}$$

The evolution of the concentrations at the outlet in t^{-1} imposes a slow-exchange dynamics, and the system continues to deliver a significant concentration of the initial compound A for a long time after offset. Given the sensitivity of gustatory chemoreceptors[4], this implies that sensory activation can still occur even at a very long time after the valve switch have replaced the stimulus with the buffer. For instance, with a tube of length $10\,cm$, inner radius $1\,mm$ and a flow rate of $10\,\mu L.s^{-1}$, we get $\tau = 15.7\,s$ and after $315\,s$ the liquid presented to the specimen would still contain 5% of the original solution. To significantly decrease the characteristic time τ, it is necessary to reduce the size of the injection channel. Reducing the channel radius ρ has a strong limitation, since at a given flowrate the bulk velocity v_0 will increase quadratically and rapidly exceed the limit of physiologically relevant values ($\approx 50mm.s^{-1}$). So if ρ decreases, q has to be decreased accordingly, such that there is no net effect on the timescale τ. The only acceptable strategy is therefore to reduce the length of the delivery channel L. By using microfluidic devices, typical values of L decrease from $\approx 10\,cm$ to $\approx 100\,\mu m$, yielding three orders of magnitude in τ. As shown in **Supplementary Figure 7-b**, in our system the onset of stimulus presentation follows the slow evolution of eq. 9 with a typical time $\tau \approx 10\,ms$.

Finally, one important asset of our microfluidic device is that it completely eliminates the stimulus in a few tens of milliseconds at injection offset, as shown in **Supplementary Figure 7-b**. This is due to the inversion of flow direction in the injection and delivery channels, which stops any arrival of the stimulus in the chamber and eliminates the stagnant volume at the outlet of the delivery channel, combined with the continuous washing of the specimen with clean buffer.

Altogether, our system allows for the delivery of pseudo-square pulses at an almost constant concentration with unprecedented steep-rising and falling edges and it allows for a complete cleaning of the specimen in a few tens of milliseconds after stimulus presentation. The high-level of control over advection offered by microfluidic systems is used here to (i) deliver several pulses of stimuli in a very reproducible fashion to the same animal, which greatly facilitates response-averaging across trials, and (ii) deliver pulses as short as $10\,ms$, which is impossible with standard techniques. Finally, as the delivery channel has an extremely short length ($100\,\mu m$) it is cleaned very fast and be used for the delivery of another chemical in a very short delay following the first stimulus.

[4]Approximately $0.5\,\mu M$ for amino-acids chemoreceptors [15].

References

[1] Jones, K. A. The palatability of amino acids and related compounds to rainbow trout, Salmo gairdneri Richardson. *Journal of Fish Biology* **34**, 149–160 (1989).

[2] Jones, K. A. Chemical requirements of feeding in rainbow trout, Oncorhynchus mykiss (Walbaum); palatability studies on amino acids, amides, amines, alcohols, aldehydes, saccharides, and other compounds. *Journal of Fish Biology* **37**, 413–423 (1990).

[3] Mackie, A. & Mitchell, A. Further studies on the chemical control of feeding behaviour in the Dover Sole, Solea solea. *Comparative Biochemistry and Physiology Part A: Physiology* **73**, 89–93 (1982).

[4] Hidaka, I., Ohsugi, T. & Kubomatsu, T. Taste receptor stimulation and feeding behaviour in the puffer, Fugu pardalis I. Effect of single chemicals. *Chemical Senses* **3**, 341–354 (1978).

[5] Mearns, K. J., Ellingsen, O. F., Døving, K. B. & Helmer, S. Feeding behaviour in adult rainbow trout and Atlantic salmon parr, elicited by chemical fractions and mixtures of compounds identified in shrimp extract. *Aquaculture* **64**, 47–63 (1987).

[6] Braubach, O. R., Wood, H.-D., Gadbois, S., Fine, A. & Croll, R. P. Olfactory conditioning in the zebrafish (Danio rerio). *Behavioural Brain Research* **198**, 190–198 (2009).

[7] Bennett, T. D. *Transport by Advection and Diffusion* (Wiley, 2012).

[8] Haynes, W. *CRC Handbook of Chemistry and Physics, 93rd Edition.* CRC Handbook of Chemistry and Physics (Taylor & Francis, 2012).

[9] Chervova, L. S. & Lapshin, D. N. The Threshold Sensitivity of External Chemoreceptor in Carp Cyprinus carpio to Amino Acids and Classical Gustatory Substances. *Journal of Ichthyology* **45**, S307–S314 (2005).

[10] Oike, H. *et al.* Characterization of Ligands for Fish Taste Receptors. *The Journal of Neuroscience* **27**, 5584–5592 (2007).

[11] Li, J. *et al.* Early Development of Functional Spatial Maps in the Zebrafish Olfactory Bulb. *The Journal of Neuroscience* **25**, 5784–5795 (2005).

[12] Mack-Bucher, J. A., Li, J. & Friedrich, R. W. Early functional development of interneurons in the zebrafish olfactory bulb. *European Journal of Neuroscience* **25**, 460–470 (2007).

[13] Yaksi Emre, Francisca, v. S. P., Niessing Jorn, Bundschuh Sebastian T & Friedrich Rainer W. Transformation of odor representations in target areas of the olfactory bulb. *Nat Neurosci* **12**, 474–482 (2009). 10.1038/nn.2288.

[14] FM, W. *Viscous fluid flow*, 123 (McGraw-Hill Book Company, 1974).

[15] Caprio, J. *et al.* The taste system a the channel catfish: from biophysics to behavior. *TINS* **16**, 192–197 (1993).
